# Effects of Material and Non-Material Rewards on Remembering to Do Things for Others

**DOI:** 10.3389/fnhum.2015.00647

**Published:** 2015-12-01

**Authors:** Maria A. Brandimonte, Donatella Ferrante

**Affiliations:** ^1^Laboratory of Experimental Psychology, Suor Orsola Benincasa UniversityNaples, Italy; ^2^Department of Life Sciences, University of TriesteTrieste, Italy

**Keywords:** prospective memory, rewards, motivation, consciousness, prosocial behavior

## Abstract

Recent research has shown that *pro-social prospective memory*, i.e., remembering to do something for others, is negatively affected by the presence of small material rewards. While this competition between pro-social and self-gain motives leads to poor memory for the intention, people do not seem to be aware of the possible collision effects of competing motives (Brandimonte et al., 2010). Extending research on this general topic, in two activity-based prospective memory (PM) experiments, we explored the effects of different types and amount of rewards on pro-social prospective remembering. In Experiment 1, participants could receive no reward, a low material reward (1 euro), or a high material reward (20 euro) for their pro-social PM action. In Experiment 2, their pro-social PM performance could be rewarded or not with an image reward (disclosure of their altruistic behavior). Results revealed that introducing a small material reward (Experiment 1) or a non-material reward (Experiment 2) impaired pro-social PM. However, introducing a high material reward eliminated the impairment (Experiment 1). Importantly, in Experiment 1, ongoing task performance in the pro-social condition was faster than in the No PM condition. However, in Experiment 2, ongoing task costs emerged in the presence of a non-material reward, as compared to the pro-social condition. Also, results from two independent ratings showed that people's predictions on their future pro-social actions were at odds (Experiment 1) or in line (Experiment 2) with actual PM performance. It is suggested that, according to the nature and amount of rewards, memory for a pro-social future action may be modulated by conscious or unconscious motivational mechanisms.

## Introduction

Remembering to do things in the future is known as *prospective memory* (PM). In the past decades, research on the cognitive mechanisms underlying PM has made enormous progress (see Brandimonte et al., 1996; Kliegel et al., 2008). However, some researchers have recently noticed (Ellis and McGann, 2005; Brandimonte and Ferrante, 2008; Altgassen et al., 2010; Brandimonte et al., 2010; Cook et al., 2015) that investigating this important aspect of everyday life only in terms of a cognitive analysis neglects the fundamental social and motivational components of this activity (see also Meacham and Kushner, 1980). Indeed, whereas there is a large body of empirical studies that investigated the cognitive processes underlying PM, only few studies so far have explored motivational PM processes. Penningroth and Scott (2007) suggested a *goal-based motivational-cognitive model* according to which motivation to fulfill an intention increases whenever an intention becomes relevant for one's personal goals, thereby influencing encoding, maintaining, and retrieving of the intention. Most recently, Cook et al. (2015) examined what they termed *value-added intentions* by manipulating the cognitive frame associated with monetary contingencies for detecting PM cues. A loss-frame was associated with a monetary punishment for failing to respond to cues while a gain-frame was associated with a monetary reward for remembering to respond to cues. Results showed that both gain and loss frames improved PM compared with a no-frame condition, and that this increase in PM is not associated with an increase in ongoing costs.

Motivation may influence the completion of intentions in various ways. It is well established that motivation directs behavior toward specific goals and may be modulated by the presence of rewards. Often, rewards increase motivation and improve cognitive performance (e.g., Pochon et al., 2002; Cook et al., 2015). However, providing a reward does not automatically and consistently increases motivation. Much depends on the nature and amount of reward, and on how the reward is perceived by the person (Locke and Braver, 2008). For instance, if the activity is one that individuals would do in the absence of rewards (see Deci and Ryan, 2000), introducing a material incentive makes people feel controlled by the reward. As a consequence, intrinsic motivation decreases and so does pro-social behavior. In the economic arena, this decrease in the quality and amount of pro-social behavior has been attributed to “motivation crowding out” and it has been documented both in field and laboratory experiments (Gneezy and Rustichini, 2000; Fehr and Fischbacher, 2003).

In the neuroscience literature, there is abundance of evidence of a strict relationship between the magnitude of the brain activation and the magnitude of the rewards (or punishments) received. However, to the best of our knowledge, only two neuroimaging studies (Murayama et al., 2010; Albrecht et al., 2014) tracked the neural correlates of motivation crowding out using functional MRI. Murayama et al.'s study provides evidence that the corticobasal ganglia valuation system plays a central role in the undermining effect. Specifically, results suggest that performance-based monetary reward indeed undermines intrinsic motivation, as assessed by the decreased number of voluntary engagements in the task (people do not feel subjective value in succeeding in the task). In particular, activity in the anterior striatum and the prefrontal areas (LPFC) decreased along with this behavioral undermining effect. These findings suggest that the corticobasal ganglia valuation system underlies the undermining effect through the integration of extrinsic reward value and intrinsic task value. Activation in the anterior part of the striatum mimics the pattern observed in the behavioral undermining effect. This supports recent psychological suggestions that the undermining effect is closely linked to a decreased sense of self-determination due to motivation crowding out.

Albrecht et al.'s (2014) study investigated the neural processes underlying the effects of monetary and verbal rewards on intrinsic motivation. Their results did not support the crowding-out effect due to monetary rewards. However, they found an effect of verbal rewards on brain activation: Activation in the anterior striatum and midbrain was higher after the administration of verbal rewards than in the control group. Once again, the activation in the striatum might reflect a higher perceived self-determination—a key component of the crowding-in effect (Deci and Ryan, 1985).

In the PM domain, much progress has been made in the last 15 years on the neural basis of PM (for a meta-analysis, see Cona et al., 2015). The majority of the studies focused on the role of the anterior prefrontal cortex (aPFC, Brodmann's area 10), which appears to play a crucial role in maintaining intention (see Burgess et al., 2011, for a review). In particular, PM conditions are associated with increased activity in lateral aPFC (BA 10) and decreased activity in medial aPFC (BA 10). Burgess et al. (2005, 2007) explained these results by proposing that lateral aPFC mediates attending to internal representations, i.e., stimulus-independent (SI) processes, such as delayed intentions for future actions. By contrast, medial aPFC is important for attending to external perceptual information, so that it mediates stimulus-oriented (SO) processes.

Interestingly for the purpose of the present studies, Cona et al. (2015) suggested that if the ongoing task is highly demanding, it would prevent individuals to be fully engaged in strategic monitoring, with the result that dorsal frontoparietal and cognitive control regions would be less involved. Momennejad and Haynes (2013) found that when the ongoing task load is high, individuals might be more likely to rely upon spontaneous processes that are governed by the reactive transient activity of insula/ventral frontoparietal network.

Despite the progress in the identification of the neural correlates of PM, research on motivation crowding-out in PM is still in its infancy and only behavioral data have been collected so far. Indeed, understanding the motivational underpinnings of memory for intentions is a complex task. Extant studies have been almost exclusively devoted to the analysis of the motivational mechanisms underlying standard PM tasks (e.g., Cook et al., 2015), i.e., memory for future actions that benefit the person who performs the action. In fact, however, often our memory for intentions involves remembering to do things for others rather than for ourselves and typically we feel more confident that we will remember to perform an action in the future if our goal has a pro-social value (Brandimonte et al., 2010). Thus, contrary to the widespread belief that people are positively motivated by reward incentives, recent studies in the economic and psychological literature have shown that performance-based extrinsic reward can actually undermine a person's intrinsic motivation to engage in a task. A recent study by Brandimonte and colleagues showed that memory for an intention can be modulated by such social variables as the direction of the benefit (for the participant or for another person) derived from the person's action and the presence of rewards (Brandimonte and Ferrante, 2008; Brandimonte et al., 2010). Namely, it has been shown that memory for an intention improves when the participant's action has a social relevance (i.e., it produces a benefit for another person). However, in accordance with social (e.g., Deci, 1975; Deci and Ryan, 1985, 2000) and economic (Gneezy and Rustichini, 2000; Frey and Jegen, 2001; Bénabou and Tirole, 2006) theories of altruism, introducing a small material reward decreases intrinsic motivation, hence impairing pro-social PM.

Notably, the potential conflict between pro-social and self-gain motives appears to operate outside of the person's awareness. To date, in Brandimonte et al.'s (2010) study, successful PM performance was accompanied by *faster* response times in the ongoing task under pro-social conditions as compared to standard conditions. In addition, when independent participants evaluated how likely they would be in everyday life to perform the pro-social action under various reward conditions, they predicted they would be *less* likely to perform the action if they were to help others *without reward* than *with reward*. However, in contrast with the ratings, performance of experimental participants in the pro-social PM conditions *was impaired* when a material reward was expected (Brandimonte et al., 2010). Taken together, these results suggest a prominent role of latent motivational mechanisms in guiding memory for pro-social intentions. On the other hand, being the first results documenting adverse effects of rewards on memory for intentions, a more subtle analysis of the specific role of rewards in activating/inhibiting pro-social PM through their interaction with intrinsic motivation is needed.

Accordingly, the purpose of the present article is to extend research on the above topic by investigating, for the first time, how pro-social prospective remembering is affected by two different classes of rewards: material rewards (money) and image rewards (disclosure of altruistic behavior). Brandimonte et al. (2010) showed that people's memory for actions that would benefit another person is *negatively sensitive* to the presence of small material rewards. However, there is much evidence supporting one of the broadest principles of behaviorism concerning how rewards may increase the frequency of our behaviors, both in everyday life and in experimental settings. For example, research on the effects of rewards on performance in standard (i.e., non-prosocial) PM tasks has typically documented better performance under material reward conditions (Meacham and Singer, 1977; Somerville et al., 1983; Cook et al., 2015). Thus, how to reconcile these two apparently opposite bulks of results? Festinger and Carlsmith (1959), in one of the most known experiments in the social psychology field, showed an inverse relationship between incentive magnitude and attitude change. Participants were asked how much they had enjoyed a boring and tedious task after they were requested to lie to a confederate saying that the task was exciting. Those who received only $1 to make the counter-attitudinal statement to the confederate rated the task as significantly more enjoyable than participants who had either received the larger sum of $20 or no incentive. Those who received 20$ solved the conflict for pro-social reasons and for self-gain reasons, while those who received only 1$ for lying, solved the conflict being congruent with their statement, as if they were *not lying*. A first question arising from those findings, which has received no answer so far, is which amount of reward makes intrinsic motivation decrease so as to defeat pro-social PM. A second question is whether pro-social PM is differentially affected by different types of reward, as it should be hypothesized on the basis of results from the neuroscience literature. For instance, more intangible rewards, such as image rewards that may affect one's own reputation, should have a differential effect on different task performance.

In the economic domain, Bénabou and Tirole (2006) suggested that although a greater prominence and memorability of contributions –such as disclosure of giving behavior- generally encourages pro-social behavior (because individuals mostly care about their social reputation), disclosure of altruistic behavior may conflict with the intrinsic motivation to help others. Indeed, for some persons, visibility of their good actions may make them become suspected of being motivated by appearances, which may reduce intrinsic motivation to remember and perform the intended pro-social action. If so, introducing a social-image reward may have a detrimental effect on motivation and, therefore, on pro-social PM.

To address the above issues, in Experiment 1 we manipulated the amount of a material reward (money) in a simple activity-based PM task (signing a form for the benefit of another person *at the end* of a highly demanding ongoing activity), so as to obtain high (20 euro), low (1 euro), and no reward conditions under socially relevant situations.

Research on the effects of rewards on performance in standard (i.e., non-pro-social) PM tasks typically documented better performance under material reward than no reward conditions (Meacham and Singer, 1977; Somerville et al., 1983; Cook et al., 2015). However, recently, it has been shown that if a *small material reward* was introduced in a PM task, it had no effect under standard PM conditions while it impaired PM performance under pro-social conditions (Brandimonte et al., 2010). In addition, in the absence of a reward, memory for the intention was better under pro-social than under standard (i.e., Non-pro-social) PM conditions. In our first experiment, we therefore expected to observe reduced pro-social PM performance under low reward conditions, as compared to both No reward and High reward conditions.

In Experiment 2, pro-social prospective behavior could be rewarded or not with an image reward (i.e., disclosure of altruistic behavior). As far as good actions become suspected of being motivated by appearances, trying to foster pro-social memory by making glory public might be a source of noise that undermines intrinsic motivation (Bénabou and Tirole, 2006), just as small material rewards do. If so, introducing an image reward should dampen pro-social PM.

Response latency in the ongoing task should also reveal important processing differences among conditions. Namely, if the effect of introducing a pro-social element to the PM task is to raise the motivational activation of completing the task at hand, ongoing task latency should decrease under pro-social conditions (see Brandimonte et al., 2010).

The resolution of the conflict between competing motives may be either consciously or unconsciously driven. If people are aware of the potential conflict between pro-social and self-gain motives, it seems reasonable to predict that when queried about how likely they would be to remember to perform the pro-social action under the various conditions, they should say they would be *more* likely to do it if they were to help others *without reward* than *with reward*. However, in Brandimonte et al.'s (2010) study, participants predicted they would be more likely to perform the action if the action produced a social *and* a personal benefit rather than solely a social benefit. This dissociation between consciously reported predictions and actual performance argues against the hypothesis that the decrease observed in pro-social PM behavior when a reward is introduced is the by-product of the person's conscious decision not to perform the pro-social action. Rather, people do not seem to be aware of the possible collision effects of competing motives.

## Experiment 1

### Methods

#### Participants and design

Eighty undergraduates from Suor Orsola Benincasa University at Naples participated in the experiment (mean age: 22.5, range: 19–28). They were randomly assigned to one of four conditions (No PM, Pro-social PM/high reward, Pro-social PM/low reward, and Pro-social PM/no reward). The No-prospective-memory condition served as control.

An additional group of 60 participants who had not taken part in the experiment were required to evaluate how important each of the pro-social situations was to them in everyday life and to predict, on a 7-point scale, how likely they would be to perform each of the three actions. The study received approval from the Ethical Committee of Suor Orsola Benincasa University. All participants provided informed consent before taking part in the experiment.

#### Procedure and stimulus materials

Procedure was based on Brandimonte et al.'s (2010) study. On arrival, the participant was informed that he/she would perform a computer-based verb identification task. There were 288 three- or four-syllables verbs, half of which were regular and half irregular. Each group of regular and irregular verbs was further divided into three subgroups of 48 verbs each, according to their ending (in Italian, ARE-ERE-IRE for the regular verbs and ARE/RRE, ERE, IRE for the irregular verbs). There were three blocks, each consisting of 48 regular and 48 irregular verbs. Within each block, the 96 items were presented twice, in random order. Overall, each participant provided 576 responses.

The ongoing task started with a fixation point that remained in view for 500 ms. Then, each verb appeared and remained in view for 5 s. The participants had to press one of two counterbalanced keys, according to whether the verb was regular or irregular. PM instructions involved remembering to sign a form at the end of each block. The form was positioned on a table out of the participant's sight. Each block was initiated by pressing any key on the keyboard. At the end of each block, the participant could either go and sign the form (if she/he remembered the PM task) or just press any key to start with the next block (if she/he forgot the PM task). Participants assigned to the No PM condition performed only the verb identification task.

To make the to-be-performed action socially relevant, the participants were told that if they will remember to sign the form, important data would be collected for the experimenter's thesis. Thus, in the PM/reward conditions participants received one of the following instructions: (a) If you'll remember to sign the form, you'll receive 1 euro (Low reward); (b) If you'll remember to sign the form, you'll receive 20 Euro (High reward). It was made clear that the reward would be provided only to those participants who signed the form all three times. A No reward condition reflected a pure pro-social PM task and served as control.

Before starting with the task, each participant was required to recall instructions, to ensure that he/she had understood *what* to do, and *when* to do the tasks. Similarly, at the end of the experimental session, each participant was interviewed again about the tasks she was supposed to do. Those participants who forgot (even for 1 time) to sign the form were also interviewed about the reasons why they did not perform the planned action as required. In so doing, we ensured that any failure to perform the PM task was not due to forgetting of the content of the to-be-performed action. Importantly, no participant knew in advance that he/she might be rewarded (none of them would therefore expect to gain a reward before starting with the experiment). In addition, care was taken to recruit each participant from different courses, so as to avoid that knowledge of the various reward conditions could affect participation and performance.

### Results and discussion

All participants recalled correctly *what* to do and *when* they were supposed to do the action. Post-experimental interviews revealed that failures to perform the action were not due to forgetting of the content of the action or to non-compliance. All participants who did not sign the form said that they *forgot* to do the action and showed regret for their failure. Results are illustrated in Table 1.

**Table 1:** **Prospective memory accuracy and ongoing response times in Experiment 1**.

	**PM task**		**Ongoing task**	
	**Mean**	***SD***	**Mean**	***SD***
No PM	–	–	2899.65	476.96
No reward	2.8	0.052	2495.69	539.71
Low reward	1.7	1.12	2551.00	580.38
High reward	2.5	1.00	2575.65	689.67

#### Prospective memory performance

The ANOVA performed on the number of correct PM responses revealed a significant effect of rewards, *F*_(2, 57)_ = 7.61, *p* = 0.001, $\eta_{p}^{2}=$ 0.21. Planned comparisons showed that, in the presence of a low reward, participants were significantly worse at performing the PM task than both under No reward, *F*_(1, 38)_ = 14.25, *p* = 0.0004, $\eta_{p}^{2}=$ 0.27, and High reward, *F*_(1, 38)_ = 7.53, *p* = 0.008, partial $\eta_{p}^{2}=$ 0.17, conditions. The difference between no-reward and high reward conditions was not significant *(F* < 1).

#### Ratings

Rating analyses were aimed at exploring whether people consciously decide that accepting a low reward in order to do a pro-social action is detrimental for their own social image and, therefore, that the most appropriate behavior is *not to do* the action if one gains a low reward for it. If so, it seems reasonable to predict that, when asked to say how likely they would be to perform the planned pro-social action in different conditions, participants will typically say that they would be more likely to remember to help others *without* being rewarded for that.

To date, participants predicted they would be more likely to perform the action if the pro-social action produced also a personal, material benefit, for themselves rather than solely a social benefit, both when they expected a low reward, *F*_(1, 58)_ = 9.49, *p* = 0.003, $\eta_{p}^{2}=$ 0.14, and a high reward, *F*_(1, 58)_ = 7.74, *p* = 0.006, $\eta_{p}^{2}=$ 0.12.

#### Ongoing task performance

##### Response times

We first tabulated the mean response time on correctly answered trials over the three blocks. Outliers (0.02%) were trimmed by removing response times that were higher than 4000 ms.

A 4 × 3 × 2 mixed ANOVA that contained the between-subjects variable of PM instructions (No PM, Prosocial PM without reward, Prosocial PM with low reward, Prosocial PM with high reward) and the within-subjects variables of Block (One, Two, Three) and Category (regular and irregular verbs) was computed. There was a main effect of Category, indicating that participants were slower at identifying irregular verbs than regular verbs *F*_(1, 69)_ = 6.66, *p* < 0.02, $\eta_{p}^{2}=$ 0.09 and an effect of Block, *F*_(2, 138)_ = 43.12, *p* < 0.0001, $\eta_{p}^{2}=$ 0.38, indicating that participants speeded up their responses through blocks. An interaction between Block and Category was found such that Block improvement was evident for regular but not for irregular verbs, *F*_(2, 138)_ = 6.91, *p* < 0.002, $\eta_{p}^{2}=$ 0.09. Planned comparisons showed that, as compared to the No PM condition, performing a prosocial PM task was associated with *faster* ongoing task performance, *F*_(1, 38)_ = 5.57, *p* < 0.03, $\eta_{p}^{2}=$ 0.13. No difference was found between any of the reward conditions and the pro-social condition (all *p*s > 0.05).

##### Accuracy

We then computed the proportion of verbs correctly identified as regular vs. irregular. These data were included in 4 × 3 × 2 mixed ANOVA which showed a significant effect of Category, confirming that, as expected, identifying regular Italian verbs is easier than identifying irregular verbs *F*_(1, 76)_ = 68.02, *p* < 0.0001, $\eta_{p}^{2}=$ 0.47. There was also an effect of Block, *F*_(2, 152)_ = 13.35, *p* < 0.0001, $\eta_{p}^{2}=$ 0.17, in that participants improved their verb identification performance through blocks. An interaction between Block and Category was also found such that block improvement was evident for regular but not for irregular verbs, *F*_(2, 152)_ = 13.99, *p* < 0.0001, $\eta_{p}^{2}=$ 0.18. There was a main effect of PM instructions, *F*_(3, 76)_ = 3.77, *p* < 0.02, $\eta_{p}^{2}=$ 0.13, indicating that participants were, in general, less accurate at performing the categorization task under pro-social PM conditions than under the No PM condition, possibly due to their speeded responses. Planned comparisons revealed that this decrease in accuracy was significant under the pro-social/no reward condition, *F*_(1, 38)_ = 9.71, *p* < 0.003, $\eta_{p}^{2}=$ 0.20. No difference was found in the ongoing task between any of the reward conditions and the pro-social condition (all *p*s > 0.05).

#### No responses

Despite the difficulty of the ongoing task, the mean proportion of no responses throughout the conditions was lower than 7%. A 4 × 3 × 2 mixed ANOVA showed no effects of PM instructions (*p* = 0.407). A significant effect of Category emerged, *F*_(1, 76)_ = 7.05, *p* < 0.001, $\eta_{p}^{2}=$ 0.09, in that the rate of no responses was higher for the irregular than for the regular verbs. The effect of Block was also significant, *F*_(2, 152)_ = 38.36, *p* < 0.0001, $\eta_{p}^{2}\;=\;0.34$.

The results from Experiment 1 confirmed that memory for the intention was significantly worse when a small material reward was introduced, hence replicating Brandimonte et al.'s (2010) results. However, it also showed that while a low reward impairs memory for pro-social intentions, a high reward leaves performance unaltered, as compared to a pro-social/no reward condition. One possible interpretation is that a financial reward always reduces any pro-social motivational effect, but the higher financial reward is motivating in its own way so that the two effects cancel out. However, according to the crowding out model (Bénabou and Tirole, 2006), effects of a high reward and crowding out effects are mutually exclusive. Crowding out effects can be predicted if and only if a *small reward* is introduced under prosocial conditions, which reduces intrinsic motivation to behave prosocially by virtue of a mechanism of reduced self-esteem, should the person accept a small reward in order to be altruistic. To the contrary, a higher financial reward is motivating in its own way and it does not crowd out intrinsic motivation, because accepting a substantial reward does not make the person feel greedy, and rather adds value to the PM action. While this remains an empirical question to test in future studies, the present results seem to support the crowding out model, as they show that only under small reward condition does PM performance drop.

The results from the ongoing task (faster performance) converge on the general idea that participants' intrinsic motivation is boosted by the pro-social nature of the task. However, as the ratings clearly show, and in line with previous results, people are not able to consciously predict their pro-social PM behavior in the presence of a material reward. To date, they predict they would be better in the pro-social PM task if they could also get a material reward, but in fact they perform worse than in the pro-social/no reward condition when the reward is a low one and as well as in the pro-social condition/no reward condition when the reward is a high one (see Brandimonte et al., 2010).

These results add important refinements to the general picture emerging from previous studies, but they also open new questions on the differential role of rewards in influencing prospective remembering. For example, it is still unknown whether rewards that involve non-material benefits (such as disclosure of the pro-social action) may determine a decrease in pro-social PM, as predicted by economic models of altruism (Bénabou and Tirole, 2006).

To explore this issue, in Experiment 2 we assessed the effectiveness of non-material rewards on participants' pro-social PM.

## Experiment 2

### Methods

#### Participants and design

Sixty undergraduates from Suor Orsola Benincasa University at Naples participated in Experiment 2 (mean age: 23.2, range: 19–29). None of them had participated in Experiment 1. They were randomly assigned to one of three conditions (No PM, pro-social PM/no image reward, pro-social PM/image reward). The No-prospective-memory condition served as control.

An additional group of 50 participants who had not taken part in the experiment were required to evaluate how important each of the pro-social situations was to them in everyday life and to predict, on a 7-point scale, how likely they would be to perform the pro-social action in each condition.

#### Procedure and stimulus materials

Procedure and materials were the same as those used in Experiment 1. Participants were all informed that their signatures were fundamental for the experimenter to complete her thesis (social relevance). The crucial modification, as compared to Experiment 1, was that the participants assigned to the image-reward conditions were also informed that if they remembered to sign the form, their name would be published in the booklet of the activities of the Laboratory of Experimental Psychology and they would be publicly thanked for their help in the next Lab meeting.

### Results and discussion

All participants but one spontaneously recalled correctly *what* to do and *when* they were supposed to do the action. This participant recalled instruction with help. Post-experimental interviews revealed that failures to perform the action were not due to forgetting of the content of the action or to non-compliance. Importantly, all the participants who *did not sign* the form claimed that they *forgot* to do the action. Results are illustrated in Table 2.

**Table 2:** **Prospective memory accuracy and ongoing response times in Experiment 2**.

	**PM task**		**Ongoing task**	
	**Mean**	***SD***	**Mean**	***SD***
No PM			2831.85	441.73
No image reward	2.7	0.66	2431.69	470.11
Image reward	1.95	1.05	2778.97	469.22

#### Prospective memory performance

A One-Way ANOVA performed on the number of correct PM responses showed a significant effect of PM instructions, with pro-social PM performance significantly worse in the presence of an image reward, *F*_(1, 38)_ = 7.33, *p* = 0.01, $\eta_{p}^{2}=$ 0.16.

#### Ratings

Participants predicted they would be more likely to perform the pro-social action if their good action was *not* made public, *F*_(1, 49)_ = 26.79, *p* = 0.0001, $\eta_{p}^{2}=$ 0.35.

#### Ongoing task performance

##### Response times

Once again, outliers (0.035%) were trimmed by removing response times that were higher than 4000 ms.

A 3 × 3 × 2 mixed ANOVA that contained the between-subjects variable of PM instructions (No PM, Prosocial PM/no image reward, Prosocial PM/image reward) and the within-subjects variables of Block (One, Two, Three) and Category (regular and irregular verbs) was computed. There was a main effect of Category, *F*_(1, 44)_ = 9.64, *p* = 0.003, *p* = 0.003, $\eta_{p}^{2}=$ 0.18 and an effect of Block, *F*_(2, 88)_ = 47.173, *p* = 0.0001, $\eta_{p}^{2}=$ 0.52. These effects were qualified by an interaction indicating that Block improvement was evident for regular but not for irregular verbs, *F*_(2, 88)_ = 9.22, *p* = 0.0002, $\eta_{p}^{2}=$ 0.17. Planned comparisons showed that, as compared to the No PM condition, performing a prosocial PM task in the absence of image reward was associated with *faster* ongoing task performance, *F*_(1, 38)_ = 11.01, *p* = 0.001, $\eta_{p}^{2}=$ 0.23. However, as compared to the pro-social condition, performing a pro-social action in the presence of image reward was associated with *slower* ongoing task performance, *F*_(1, 38)_ = 7.64, *p* = 0.009, $\eta_{p}^{2}=$ 0.17. There was no significant correlation between ongoing RTs and PM performance across conditions (all *p*s > 0.05).

##### Accuracy

A 3 × 3 × 2 mixed ANOVA showed a significant effect of Category *F*_(1, 57)_ = 73.86, *p* < 0.0001, $\eta_{p}^{2}=$ 0.56. There was also an effect of Block, *F*_(2, 114)_ = 6.40, *p* < 0.001, $\eta_{p}^{2}=$ 0.10, and an interaction between Block and Category, *F*_(2, 114)_ = 12.23, *p* < 0.0001, $\eta_{p}^{2}=$ 0.18. There was a main effect of PM instructions, *F*_(2, 57)_ = 6.27, *p* < 0.02, $\eta_{p}^{2}=$ 0.18, indicating that participants were, in general, less accurate at performing the categorization task under PM conditions as compared to the No PM condition. There was no significant correlation between ongoing accuracy and PM performance across conditions (all *p*s > 0.05).

#### No responses

A 3 × 3 × 2 mixed ANOVA showed no effects of PM instructions (*p* = 0.215). A significant effect of Category emerged, *F*_(1, 57)_ = 8.30, *p* = 0.005, $\eta_{p}^{2}=$ 0.13, in that the rate of no responses was higher for the irregular than for the regular verbs. The effect of Block was also significant, *F*_(2, 114)_ = 31.58, *p* < 0.0001, $\eta_{p}^{2}\;=\;0.36$.

The results from Experiment 2 showed that pro-social PM is negatively affected by a non-material reward just as it is impaired by a small material reward (Experiment 1). However, the results from the ongoing task provide a more complex picture. Ongoing task performance under the pure Pro-social (i.e., No image reward) condition showed faster response times, as compared to the No PM control condition, hence replicating results from Experiment 1. In contrast, ongoing task performance under the Pro-social/Image reward condition showed *slower* response times as compared to the control Pro-social condition, hence revealing costs (Smith, 2008) in the presence of an image reward^1^. Coherently, the ratings indicated that people are indeed able to consciously predict their pro-social PM behavior (i.e., they predict they would be worse) in the presence of a non-material reward. However, the lack of correlation between ongoing and PM performance suggests that such costs were not due to the fact that participants have directed resources away from the ongoing task and toward the PM task.

## General discussion

Remembering to do things *for others* is at the heart of a person's social life as often our memory for actions is aimed at providing some benefits to other individuals. Yet, the mechanisms underlying pro-social PM are still almost totally unknown. The present article therefore explored whether pro-social prospective remembering is influenced by the amount and the type of rewards, using behavioral measures and new paradigms that, with appropriate modifications, might prove useful for future studies with neuroimaging techniques.

Overall, the present results added important refinements to the picture emerging from previous research by showing that, under socially relevant conditions, introducing a small material reward (money) or a non-material reward (disclosure of altruistic behavior) dramatically decreased memory for the intention, whereas a high material reward left pro-social PM performance unaltered.

In the present research, we documented, for the first time, a decrease in pro-social prospective remembering following the introduction of an image reward. This new finding may be rather counterintuitive. After all, most pro-social behaviors in everyday life seem to positively depend on disclosure of the giving behavior. However, it seems equally plausible that if people are mostly concerned about not appearing a double-faced person, introducing a social-image reward may have a detrimental effect on motivation and therefore people may not feel motivated to comply with the pro-social task if they are rewarded for that (Gneezy and Rustichini, 2000). Indeed, as far as good actions become suspected of being motivated by appearances, trying to foster pro-social memory by making glory public may be embarrassing for the person. Cognitive evaluation theory (CET; Deci and Ryan, 1985) predicts that rewards only reduce intrinsic motivation when they are perceived as controlling the behavior, and might actually enhance intrinsic motivation if they engender feelings of competence. Whether rewards undermine the initial “intrinsic” (not narrowly self-interested) motivation for the targeted behavior may therefore depend on whether the reward is seen as an attempt to “purchase” the behavior from the individual.

Such an interpretation is supported by the present results. In particular, analyses of the ongoing task performance provided a rather consistent picture. To date, in Experiment 1, ongoing task performance in the pro-social condition was faster than in the control condition. In contrast, in Experiment 2, ongoing costs emerged in the presence of the image reward, as compared to the pro-social condition. The lack of correlation between ongoing and PM performance might indicate that the slowing down of response times reflects attentional costs due to the participants' motivation of not appearing a double-faced person, rather than attentional costs due to the fact that participants have directed resources away from the ongoing task and toward the PM task. This interpretation is strengthened by the fact that the activity-based PM task was very simple and could be performed without any ongoing cost (Experiment 1, see also Brandimonte et al., 2010).

Consistent with social and economic models of pro-social behavior and with recent neuroimaging research (Murayama et al., 2010), these results hint to a conclusion in terms of intrinsic motivation vs. extrinsic motivation as important determinants of human behavior, with intrinsic motivation susceptible of being modulated by rewards, hence affecting pro-social PM. In particular, while these studies showed, once again, that intrinsic motivation is susceptible of being undermined by reward, it is also clear that such rewards do not need to be material ones, as both material and non-material rewards impaired PM performance. In addition to crowding out effects, these results highlight important processing differences in the way material and non-material rewards interact with intrinsic motivation. To date, while both small and high material rewards appear to modulate motivation (though in opposite directions) by operating outside of the person's awareness, introducing an image reward appears to produce motivation crowding out through a conscious process, as indicated by the ongoing task analyses and by the results from the ratings (see also Brandimonte et al., 2010).

While we know nothing on the neural correlates of pro-social PM under different reward conditions, a reversed inference, from extant neuroimaging results to plausible predictions of the present studies, can be attempted. For instance, increased level of tonic activity in the network of brain regions including the right lateral, the right parietal cortex, the dorsal medial frontal cortex, and the left cerebellum might predict motivation-related changes in pro-social PM performance due to the introduction of different amounts of rewards, as observed in the present experiments. In particular, as documented by Murayama et al. (2010), decreases in the activity of the anterior striatum and the prefrontal areas (LPFC) should be related to behavioral undermining effects observed in our experiments with small monetary rewards, whereas, as documented by Albrecht et al. (2014), activation in the striatum might be related to the better performance observed under high amount of monetary reward, hence reflecting a higher perceived self-determination. From our results, one might also speculate that, despite crowding out effects occur with both types of reward, the differences in the ongoing task response times that we observed between material and non-material rewards (longer ongoing task response times in the non-material reward condition) might be differentially related to the activity of the anterior striatum and the prefrontal areas, with a moderate decrease in those areas in the presence of non-material rewards and a higher decrease in the presence of a material reward. Last but not least, extant results from neuroimaging studies of PM seem to fit well with the present results. Indeed, the striking differences observed in the pattern of RTs and ratings between our experiments might reflect differences in the degree of spontaneous retrieval guided by material vs. non-material rewards, in accordante with the Multiprocess view (McDaniel and Einstein, 2000, 2007) and the Gateway Hypothesis (Burgess et al., 2007).

Despite the above reasoning is speculative and relies on reverse inference, we, nonetheless, believe it might be useful for guiding future research.

In conclusion, the present results highlight the theoretical significance of considering the interaction between motivational and cognitive mechanisms when investigating memory for delayed intentions in social contexts and call for a deeper investigation of this phenomenon with neuroimaging techniques.

### Conflict of interest statement

The authors declare that the research was conducted in the absence of any commercial or financial relationships that could be construed as a potential conflict of interest.

## Footnotes

^1^Commonly, monitoring costs are measured by comparing conditions with a PM task with conditions without a PM task. However, this is appropriate only when the only change between the two conditions is the presence of a PM task. In the present research, especially on the basis of hypotheses, the most appropriate comparisons were pro-social PM vs. No PM conditions, on the one hand, and pro-social PM vs. pro-social/reward conditions, on the other hand.
